# One-Time URL: A Proximity Security Mechanism between Internet of Things and Mobile Devices

**DOI:** 10.3390/s16101694

**Published:** 2016-10-13

**Authors:** Antonio Solano, Raquel Dormido, Natividad Duro, Víctor González

**Affiliations:** Departamento Informática y Automática, ETSI Informática, UNED Juan del Rosal 16, Madrid 28040, Spain; raquel@dia.uned.es (R.D.); nduro@dia.uned.es (N.D.); vgonzalez165@alumno.uned.es (V.G.)

**Keywords:** Internet of Things, Web of Things, cloud computing, mobile communication systems, pervasive computing, authentication, web-based services

## Abstract

The aim of this paper is to determine the physical proximity of connected things when they are accessed from a smartphone. Links between connected things and mobile communication devices are temporarily created by means of dynamic URLs (uniform resource locators) which may be easily discovered with pervasive short-range radio frequency technologies available on smartphones. In addition, a multi cross domain silent logging mechanism to allow people to interact with their surrounding connected things from their mobile communication devices is presented. The proposed mechanisms are based in web standards technologies, evolving our social network of Internet of Things towards the so-called Web of Things.

## 1. Introduction

Internet of Things (IoT) emerges as a paradigm to connect things to the Internet providing a bridge between the physical and digital worlds [1]. To this end, the uniform resource locator (URL) arises as an easy and pervasive way of using web technologies to point us towards the virtual representation of things in the Internet, called in this paper digital avatars.

Short-range radiofrequency technologies like near field communications (NFC), Bluetooth low power (BLE) or WiFi, are becoming pervasive technologies on our smartphones [2,3]. Such radiofrequency technologies allow us to discover URLs on the move and automatically initiate Internet connections by providing a physical web browsing experience. Some well known examples of how to facilitate access to specific web pages by using short range radio frequency technologies are:

- smart posters with passive NFC tags [4],
- iBeacons [5] technologies in brick and mortar stores,
- welcome web pages on free WiFi hostpots.

But how do we guarantee that we are accessing the URL of a virtual representation of a thing only when we are in proximity? Short-range radio frequency technologies facilitate the discovering of URLs, but do not guarantee we are located close to the associated thing. For example, the URLs can be retrieved from the browser’s history on a mobile.

In this paper, a mechanism to tokenize URLs which are broadcast through open hardware devices [6] by using short-range radio frequency technologies is proposed. To this end, an enhanced approach based on one-time URLs that are “consumed” after usage is presented. In this approach, things are represented by dynamic URLs, which either will change once they are accessed or they will be renewed after a predefined timeout. As a result, reading this URL from a mobile phone will become a proof of close physical proximity. The tokenized URL is uniquely associated with the thing, which can be easily discovered by a smartphone enabled with short-range radio frequency technologies. If a user reopens the URL from the browser’s history, and the proximity cannot be guaranteed by other means, the connection will be rejected.

The dynamic URLs are sent from the server to the connected things. These dynamic URLs are generated in a server on the cloud and are usually broadcasted by resource-constrained devices, such as UriBeacons (http://www.uribeacon.org), Dynamic NFC tags or peer to peer (P2P) NFC modules, which become electronic components of our connected things. It is necessary to implement a security mechanism to avoid any hacker from spoofing the URL and using it, or perform man-in-the-middle (MiM) attacks by changing the original URLs and redirecting us to fishing websites that are infected with malware. In this paper, an encryption mechanism to protect key information while using very limited and constrained electronic devices that do not allow us to apply standard HTTPS mechanisms to secure all information exchanged online is also proposed [7,8].

On the other hand, to fully interact with our nearby social things [9], it is not enough to discover the URLs of the things with our smartphone. At some point in time, it is possible that we may have to allow the thing to know who we are. Authentication is the process of verifying that “someone is who he claims to be”, and this is usually carried out via credentials: username and password. Following the authentication, it is the authorization to grant the thing access to end-user profile and/or vice versa. In order to guarantee a good end-user experience, the proposed design uses the minimal number of interactions to register and establish a dialogue between the user’s smartphone and the thing. A silent logging mechanism based in Cross Origin Resource Sharing (CORS) (http://www.w3.org/TR/cors/) and LocalStorage (http://www.w3.org/TR/webstorage) techniques, both available in HTML5 (http://www.w3.org/TR/html5/), is also introduced in this paper. The first time the user access the system, this mechanism will encrypt and store the user credentials on the browser’s memory. Next time the user initiates a browsing session towards any of the URLs published from our solution, silent logging process will happen in background and the links between our mobile smartphone and our nearby connected things will be established automatically, allowing us interact with the things through the Internet. The extensive usage of web technologies in our work, is our main contribution to so-called Web of Things (WoT).

All these introduced mechanisms have been implemented to provide a mobile payment for unattended point of sale (PoS). Prior to deliver the goods or services, end-user proximity to PoS has to be verified and this is in fact the main challenge to be accomplished in our work.

To these ends, the paper is organized as follows: Section 2 introduces the motivation of presented work and a picture of our open hardware setup is included. Section 3 presents the end-user scenarios based on pervasive mobile short-range radio frequency technologies and suggests cost-effective devices to publish dynamic URLs for each respective technology. Section 4 describes the life cycle of tokens used to generate dynamic URLs. In Section 5, the proposed architecture based on cloud computing and open source software is presented. Section 6 is devoted to analyze in deep the security issues. Section 7 aims to clarify all mechanisms presented with a call flow scenario. Section 8 provides some performance benchmarks of our open hardware prototype. Finally, Section 9 summarizes the principal contributions developed in this paper.

## 2. One-Time URL Motivation

**Figure 1:**
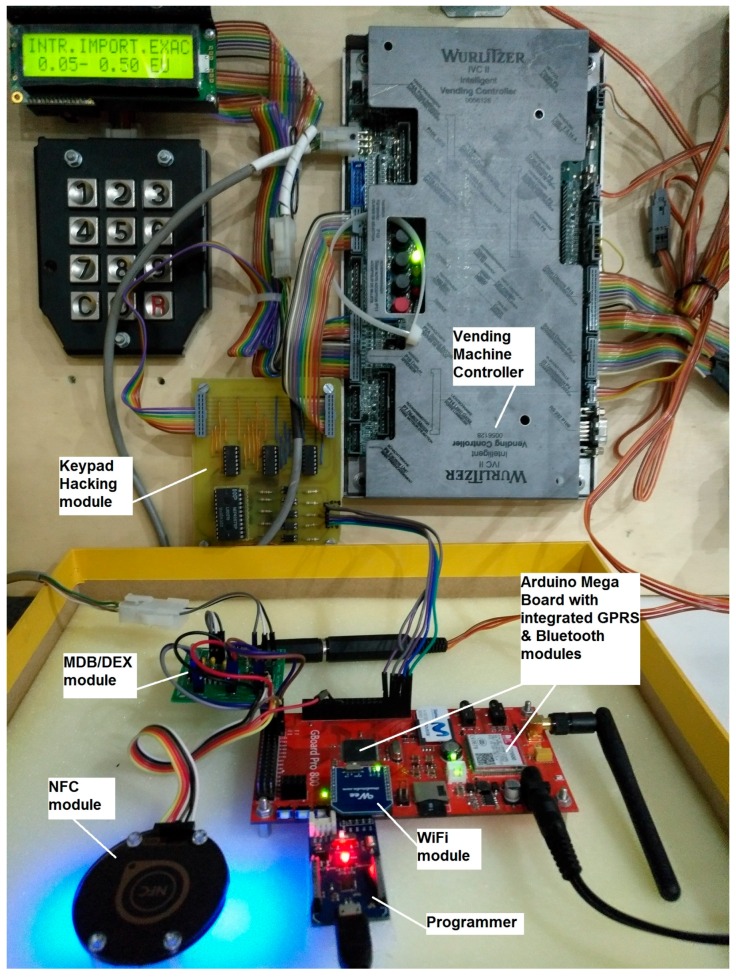
Open hardware prototype.

The ultimate goal of our research is to provide an alternative mobile payment for unattended point of sales such as vending machines but it is applicable to toll gates, parking lots, etc. Similar to the Physical Web Google approach [10], each connected vending machine is identified by means of a URL. The URL points to a Web application (webapp), which is in fact the digital avatar of the vending machine. By accessing this URL from a smartphone, consumers will be able to select, from the touchscreen of their smartphones, any of the products of the vending machine and pay for it. Immediately the ordered product will be dispensed by the vending machine. Figure 1 shows an Arduino open hardware prototype to achieve the described mobile payment scenario.

All modules highlighted in Figure 1 are on-the-self products except the MDB/DEX module (multi-drop bus/digital exchange module) and the Keypad Hacking module. These two printed board circuits (PBCs) have been designed for interacting with vending machines in a fully contactless way, i.e., without physically touching the vending machine. This contactless innovation requires implementing a mechanism to guarantee that the consumer is physically close to the vending machine when products are ordered and dispensed. Collocate the end user and the vending machine, at the time the transaction occurs, is the challenge addressed in this paper. This challenge is solved if the only way to access the digital avatar of the vending machine is through the dynamic URL, and if this URL can only be consumed from the smartphone of an authorized end user.

From the end-user perspective, the presented approach based on URLs changes completely the paradigm of having multiple native applications (apps) installed on smartphones which have to be downloaded previously. In the next section, how to interact with nearby things without needing the installation of native apps is presented. As a key competitive contribution, this paper demonstrates that it is possible to provide webapps based on HTML5 with similar functionality than native apps. Indeed, as described in Section 6.2, the end-user purchasing experience is enhanced providing a universal method to grant access to any service provided by the system.

## 3. Proximity Scenarios Based on Short Range Wireless Technologies

The aim of this section is to define a user-friendly scenario for mobile users to discover and interact with connected things based on pervasive radio-frequency technologies available on smartphones such as WiFi, Bluetooth or NFC. 

**Figure 2:**
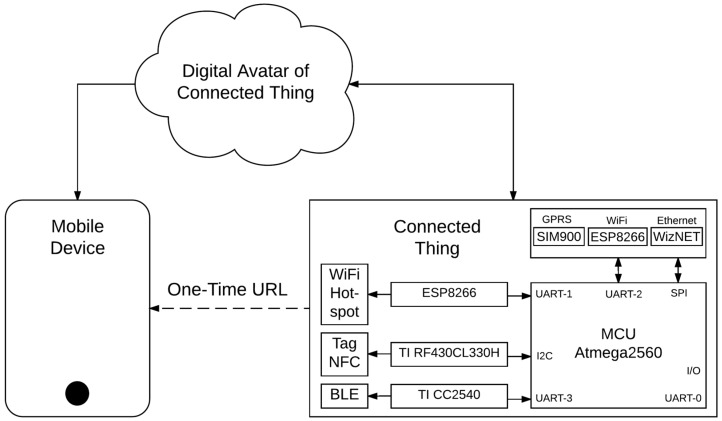
Short range wireless technologies available on smartphones.

In the following subsections, these three short-range radio-frequency technologies are introduced to achieve the same scenario: initiating a mobile browsing session to a landing web page to interact with our surrounding connected things. Figure 2 despites how to add communication modules to a simple 8 bits microcontroller.

### 3.1. The Tap & Go Reference Model

Near field communications (NFC) is a radio-frequency identification (RFID) technology operating at 13.56 MHz that enables communication between devices that are held in close proximity. NFC forum introduced their first standardized technology architecture and standards for NFC compliant devices in June 2006. 

NFC devices may be active or passive. A passive device, such as an NFC tag, contains information that other devices can read but does not read any information itself. An active NFC device, like a smartphone, would not only be able to read NFC tags, but configured in peer to peer (P2P) mode, it would also be able to exchange information with other compatible phones or devices and could even write or alter the information on the NFC tag, if authorized to make such changes. A NFC tag is a passive device with an EEPROM (electrically erasable programmable read-only memory) memory attached to the RFID inlays of the antenna. The power the tag needs to operate and communicate the information stored in the EEPROM, comes from the electromagnetic field generated by the emitter, usually a smartphone acting as NFC reader.

A dynamic NFC tag features an SRAM (static random access memory) bank that can be also accessed through a low-power Inter-Integrated Circuit (I²C) interface and therefore easily be overwritten from any low cost microcontroller. A NFC-enabled smartphone, by default, is configured to initiate P2P communications or to read NFC tags. Therefore taping a dynamic NFC Tag with a recorded URL will initiate a browsing session on the mobile device. For the proof of concept of our research we have implemented prototypes based on the cost-effective RF430CL330H chipset from Texas Instruments (Dallas, TX, USA) as it is shown in Figure 1 and Figure 2.

### 3.2. The Beacon Reference Model

iBeacon is a protocol standardized by Apple (Cupertino, CA, USA) and introduced in 2013. iBeacons uses Bluetooth low energy (BLE) proximity technology to broadcast their identifier to notify nearby devices of their presence. The technology enables smartphones, tablets and other devices to perform actions when in close proximity to an iBeacon.

In October 2014 Google (Mountain View, CA, USA) created UriBeacon, an open specification to connect Bluetooth low power beacons to the Web. Instead of broadcasting an identifier as iBeacon does, UriBeacons broadcast a URL. In 2015, UriBeacon evolved to become part of the Eddystone-URL (https://developers.google.com/beacons/), which was launched by Google in July 2015.

The TI CC2540 (Texas Instruments, Dallas, TX, USA) is a cost-effective, low-power, true system-on-chip (SoC) for Bluetooth low energy applications used in our open hardware prototypes as it is shown in Figure 2.

### 3.3. The Free Hotspot Reference Model

Welcome web pages are commonly used when connecting to free WiFi hotspot. Nowadays we can also find cost-effective system-on-chip (SoC) for WiFi, for example the ESP8266 module, which may be used to create a bespoke hotspot as shown in Figure 1 and Figure 2.

### 3.4. Alternative Models Not Based on Short Range Wireless Technologies

It is worth mentioning an alternative mechanism to read URLs using a smartphone´s cameras. Dynamic URLs can be easily encoded on quick response (QR) codes or augmented reality (AR) makers that can be displayed on screens. But nowadays, cameras on smartphones are not natively processing the images to discover the QR codes or AR markers. So, it is necessary to use an application to performance these actions. In a future, if these technologies based on image and patterns recognition are becoming pervasive technologies on smartphones, the presented approach on this paper should also apply.

## 4. Defining the Live Cycle of Dynamic URLs

**Figure 3:**
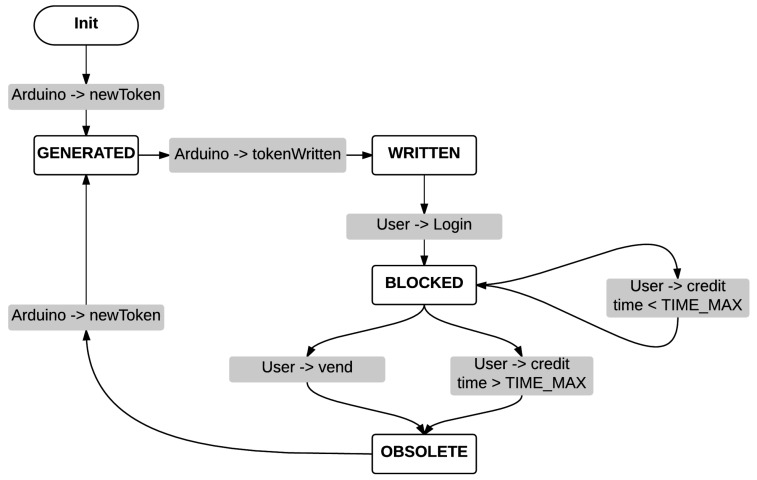
Token life cycle.

The main idea behind our research goal is that one-time tokenized URLs, which are announced by connected things, may be used to determine the physical proximity of mobile communication devices. To tokenize a URL is just enough to add a token as parameter to the URL. The Figure 3 depicts the life cycle of the token. 

As it is shown, the life cycle of the token follows a state machine workflow in response to events coming either from the connected thing or from the end user mobile device. Furthermore, this approach allows to detect unusual situations and to implement easily corrective actions. For example, if there is a communication problem with the connected thing, the token in the server will be different than the one stored in the NFC tag. At this point, a cross-platform two-factor authentication mechanism may be introduced as it will be described in Section 6.3. Tokens can be found in the following states:

- GENERATED: The token has been generated and sent to the connected thing but no confirmation of dynamic URL publication is yet received.
- WRITTEN: Connected thing has positively confirmed that the token has been written, for example, on the NFC tag.
- BLOCKED: A situation to avoid if a request for a new token arrives to the server while the end user is still interacting with the connected thing. For example, when a user initiates an e-commerce transaction with a connected point of sale, the current token (which will be in WRITTEN status) will change to BLOCKED state, not accepting new token requests until the end of the transaction.
- OBSOLETE: If a logged user does not finish the expected action or does not log out after a reasonable time, the token expires and becomes obsolete.

## 5. Underlying Cloud Based Architecture

**Figure 4:**
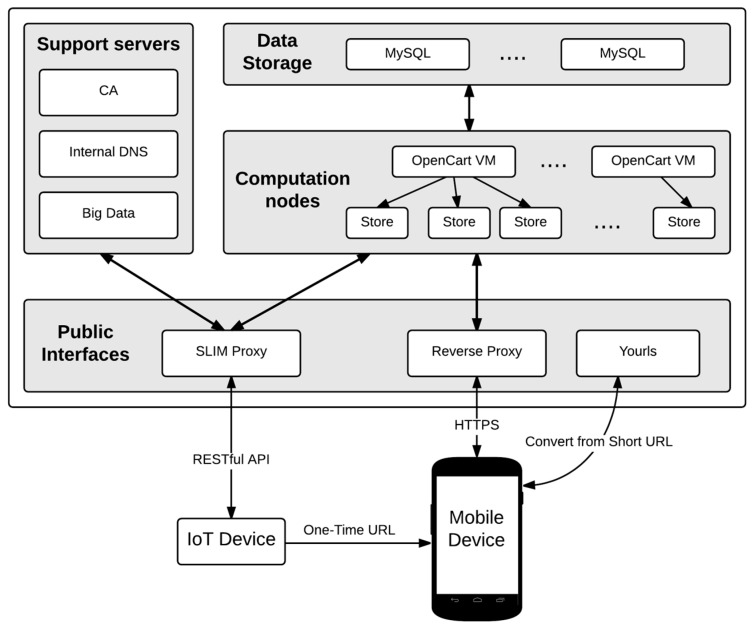
Cloud based architecture.

Recently, many research works are interested in combining Cloud Computing and IoT [11]. Figure 4 shows our end-to-end cloud based architecture. The approach is similar to the one followed in [12], but it benefits from the proximity-layer security mechanism presented in this paper.

The overall infrastructure consists of a private cloud, which uses COTS (Commercial Off-The-Shelf) bare-metal servers running OpenStack (https://www.openstack.org/) open source. A detailed description of the main building blocks are presented in the following subsections.

### 5.1. Slim Proxy

This is the interface to connect things with the platform. It is based on SLIM (http://www.slimframework.com/), a Representational State Transfer (RESTful) open source framework. It provides a REST-based interface to exchange JavaScript Object Notation (JSON) messages between IoT devices and the platform. The main roles of this server are described below.

#### 5.1.1. Support a Plug-and-Play Mechanism

The very first time a physical object is connected, an ApiKey identifier is created and linked with the corresponding digital avatar. This digital avatar will be instantiated by the platform and being accessible through the aforementioned dynamic URL.

The process of building a digital avatar, by means of a virtual machine instance in OpenStack, consists of a series of steps executed in a specific order. The details of such plug-and-play mechanism can be found in [13].

#### 5.1.2. Route Communications between Things and Their Digital Avatars

The SLIM proxy enables a translation table to map ApiKeys with assigned floating IPs in OpenStack. The ApiKey header is embedded in every HTTP request defined as a RESTful interface. The JSON messages are found in the body of the RESTful interface. JSON messages will be routed from IoT devices to the right floating IP using the translation table and finally reach the virtual representation of the thing.

### 5.2. Reverse Proxy

On the other hand, in the proposed design, the fact of having to virtualize potentially any physical object requires that each connected thing has its own URL. Using different domains or subdomains could be an option. For example, https://mything.mydomain.com.

This option has to be configured in public Domain Name Servers (DNS) and requires some synchronization time before the URL can be reached from the Internet.

For the sake of simplicity the proposed approach defines URLs for things based on a domain followed by a unique path to address the virtual representation of the thing. For example, https://mydomain.com/mything.

A standard Apache reverse proxy is used to route the URLs to the right floating private IPs and private subdomains where reside the web servers that serve these URLs. However, this system has as drawback that requires a script to be running during the initialization of the virtual machines in OpenStack as described in [13].

### 5.3. URL Shortener

This service is required for the tokenization of the URL and for the verification of physical proximity between connected things and mobile devices, which is our main contribution in this paper. Other motivations to introduce a shortener in the proposed design were to reduce the payload in the communications with our IoT devices and therefore the memory required to store and broadcast dynamic URLs.

Our solution is based on an open source called YOURLS (http://yourls.org/), which stands for Your Own URL Shortener. It is a small set of PHP (hypertext processor) scripts that allows running our own URL shortening service. The internal architecture of a shortener is quite simple. Any URL of any size generates a fixed size code that is stored in the database and it is associated with the URL. Some shorteners allow choosing the short code while others simply generate a pseudorandom sequence of fixed size. In our design, the generated tokens are pseudorandom sequences of 36 characters that are added to the URL, for example, https://mydomain.com/myoperator/mything/?route=module/openvend&token=HPLoMLNzQuQdVFe-wSv9Pg.

YOURLS codes the long URLs in a sequence of six characters by taking the first characters of the generated token. As an outcome of this process, the SLIM server composes a JSON message, like {“u”: “HPLoML”}, which will be populated to the IoT device to compose a short URL for example, https://myshortener.cc/HPLoML.

When YOURLS server receives this request, it extracts the short code “HPLoML” and it searches in its database for the long URL that corresponds to this code. Then, it performs a redirection to the page pointed by this URL in a completely transparent manner for the end user. YOURLS has many advantages, but the most determining factors when selecting it for using on this work are the following:

- It is a free and open source, which is a prerequisite for all software used in our design.
- Easy installation on our own private cloud, without limits on number of shortened URLs.
- It generates sequential keys or allows customized keys, allowing us to generate keys randomly by a hash function.
- Availability of plugins to enhance functionality. In our design, it is necessary to eliminate short URLs from the database once they have been used (as they are for one time use). This feature is not available in the default installation of YOURLS, but can be added using the plugin API (application programming interface).

### 5.4. Other Servers

In previous sections the key servers needed in our architecture to implement the proposed proximity mechanism have been detailed. In this section it is introduced, at a very high level, the rest of servers presented in Figure 4 to despite the overall architecture. These are the following:

- Application Web Servers: They provide the virtual representation of the connected things. As shown in Figure 4, OpenCart (http://www.opencart.com/) is the open source selected in our real deployment to virtualize unattended point of sales.
- MySQL (https://www.mysql.com/) Servers: This is a good design practice in order to dissociate the compute nodes and the data storage nodes in a Cloud infrastructure based on OpenStack.
- Certificate Authority (CA): Security requirements force to perform communications using HTTPS which require digital certificates in our servers. In order to maintain a low-cost system we create our own Certificate Authority using OpenSSL (https://www.openssl.org/).
- DNS Server: Currently URL addressing is done through the reverse proxy, but to provide high scalability, an internal DNS it is considered in the architecture. The selected open source is BIND (http://www.isc.org/).
- Big Data: One of the new areas being followed now in this research project is the massive analysis of the data generated. To do so, we will require new servers whose task will be the collection and analysis of all transactions taking place on the platform.

## 6. Security by Design

Designing a secured proximity layer system to interact with connected things from mobile devices requires the analysis of security from different perspectives as described below.

### 6.1. Unauthorized Users: Encryption of User Credentials on Smartphones

To guarantee a good end-user experience our design requires a minimum number of interactions between the user and the terminal. Unfortunately this requirement collides with the security requirements.

In order to achieve this goal, the local Storage of the HTML5 web browser is used to store the user credentials to allow a silent logging mechanism. The drawback of this solution is that anyone who has access to the smartphone can get these credentials unless they are encrypted. The solution includes the concept of one time token which is consumed after each logging as described below.

**Figure 5:**
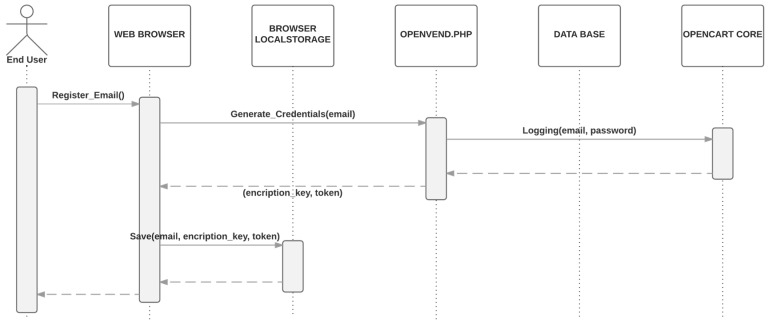
Registration process with end-user email and one-time token.

**Figure 6:**
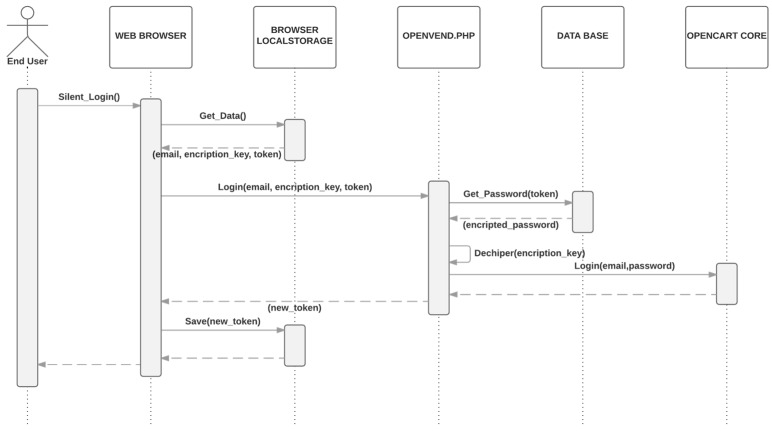
Silent logging process and credentials tokenization.

1. The username and password are stored in the server encrypted.
2. The encryption key is stored in the localStorage of the smartphone browser, along with one-time token generated by the server. Figure 5 is a simplified sequence diagram of the registration process which only requires as input a valid end-user email.
3. Next time the user is going to perform a purchase, a silent login mechanism will retrieve the credentials as show in Figure 6 Both, the encryption key and the token, are sent to the server. The token is checked and if it is correct, the key is used to decrypt the user credentials to log into the server. The encryption key is not kept in the server after it is used.
4. A new token is generated and sent to the user’s smartphone.

The advantage of this mechanism is that, if the smartphone is compromised, the attacker can only get access to the key to decipher the credentials, but not to the credentials themselves (which are stored and encrypted in the server). The one-time token guarantees that if an attacker gets the key, she/he can only access the system once, but still, this access can be identified as an intrusion and be notified to user´s email. A functional demo to show the registration process and silent logging mechanism is available on [14].

### 6.2. Cross Origin Resource Sharing (CORS)

For security and privacy reasons Web browsers prevent documents in different domains from affecting each other; that is, cross-site scripting is disabled. While this is an important security feature, it prevents pages from different domains or subdomains access to data stored on the local storage of the HTML5 web browser. In Section 6.1 how to store the user credentials encrypted on the localStorage with the aim to have a silent logging mechanism for all connected things is described, but if connected things are addressed by URLs using subdomains, as OpenCart internally does, the silent logging mechanism will not work properly. The method used to circumvent this problem is based in CORS, a cross-document messaging HTML5 API that provides a mechanism that allows the interchange of messages between documents hosted in different domains or subdomains.

The main idea behind CORS is to create an HTML document hosted in a public domain, such as the one configured in our SLIM proxy that acts as a server. The only job of this document is to handle all the operations related to the localStorage. Every webapp, regardless of the domain in which it is hosted, sends a message to the server requesting the read or write operation over the localStorage and the server is the one that performs it, returning to the webapp the result of the operation. This means that the owner of the localStorage is the domain that hosts the server document and the only way to access it is through the server document.

In order to avoid unauthorized requests from other domains, the server stores a whitelist of domains that are allowed to perform operations in the localStorage. The other file needed for the implementation of CORS provides the JavaScript supporting the functionality needed in the webapp side. Its first task is to create an HTML iframe that points to the server file.

Once this operation has been done, this file provides the functions to read and write in the localStorage. These functions, invoked from the webapp, resend the request to the server file using the cross-document messaging. That is, the function is called from a webapp hosted in any domain included in the whitelist, but it is redirected to the server which is the owner of the localStorage and the one that performs the operations over it.

In other words, using both the CORS mechanism and the silent logging mechanism presented in Subsection 6.1, results in the possibility to have a whitelist of URLs in different domains which may be accessed by the same end user. For instance, if domain A belongs to a service provider and domain B belongs to a second service provider, the end user will access both domains if previously service providers agreed to share the end-user credentials. Potentially, the presented work provides a universal method to grant access to any service provided by the system.

### 6.3. Enhancing Security with a Cross-Platform Two-Factor Authentication Mechanism

The silent logging and dynamic URL mechanisms are happening in the background to enhance the end-user experience, but sometimes it may be required to request explicit actions to end user, for example when performing in-store high value ecommerce transactions or when dynamic URLs are corrupted.

On such scenarios additional proximity mechanism may be implemented at check-out time. For instance, a personal identification number (PIN) may be generated randomly for each transaction and shown in the display of the connected point of sale. The end user has to enter this PIN into the webapp to validate the transaction. 

This mechanism reinforces the collocation of the user with our nearby-connected things, but also permits the usage of other “discovery” technologies that use static URLs such as: quick response (QR) codes (see demo [14]), augmented reality (AR) markers, webapp shortcuts on mobile devices, etc. 

Alternatively, the presented two-factor authentication mechanism can be used to pre-order products remotely on unattended point of sales. In such scenarios the consumer will receive a PIN code on his mobile phone. To dispense the pre-ordered product, consumer only has to type the PIN on the keypad of the unattended point of sales, which becomes a proof of the consumer´s presence.

### 6.4. Secure Web Communications from Mobile: Adding Secure Socket Layer (SSL)

As our main interface to interact with the thing is a smartphone provided with a modern web browser, the digital representation of the thing will be in the form of a webapp. Nowadays, from a end-user experience perspective, webapps are at same level of native apps for many scenarios thanks to HTML5 standards. Our webapps use HTTPS to secure all information exchanged online.

### 6.5. Secure IoT Communications: Following Lightweight Machine-to-Machine (LWM2M) Security Model

To secure the IoT device communications, our design is guided by LWM2 (http://openmobilealliance.hs-sites.com/lightweight-m2m-specification-from-oma) recommendations and uses pre-shared keys (PSKs). PSK is preferred to other mechanisms also proposed by LWM2M for very constrained devices with limited computing and memory resources. This design decision implies that the same PSKs and PSK IDs need to be generated and installed on the IoT device and on the backend. LWM2M proposes two modes:

- TLS_PSK_WITH_AES_128_CCM_8,
- TLS_PSK_WITH_AES_128_CBC_SHA256.

Both options provide all the requirements of a cryptographic system: confidentiality, authentication and data integrity. In our proposal it is selected the second mode considering the availability of open source libraries from Arduino (https://www.arduino.cc/) community. Table 1 shows some benchmarks of test performed with an Arduino Mega board and open source libraries detailed in ANNEX 1.

When a message is encrypted the result is a random sequence of bits. This result has to be encoded into printable characters to be sent in the message. There are two options for this: hexadecimal or American Standard Code for Information Interchange (ASCII) characters. Hexadecimal encodes each byte of information as two bytes. ASCII uses the Base64 encoding where 3 bytes are encoded in 4 bytes. Due to the resource-constrained memory of our IoT devices the Base64 encoding (lower overhead) has been selected in our design.

**Figure 7:**
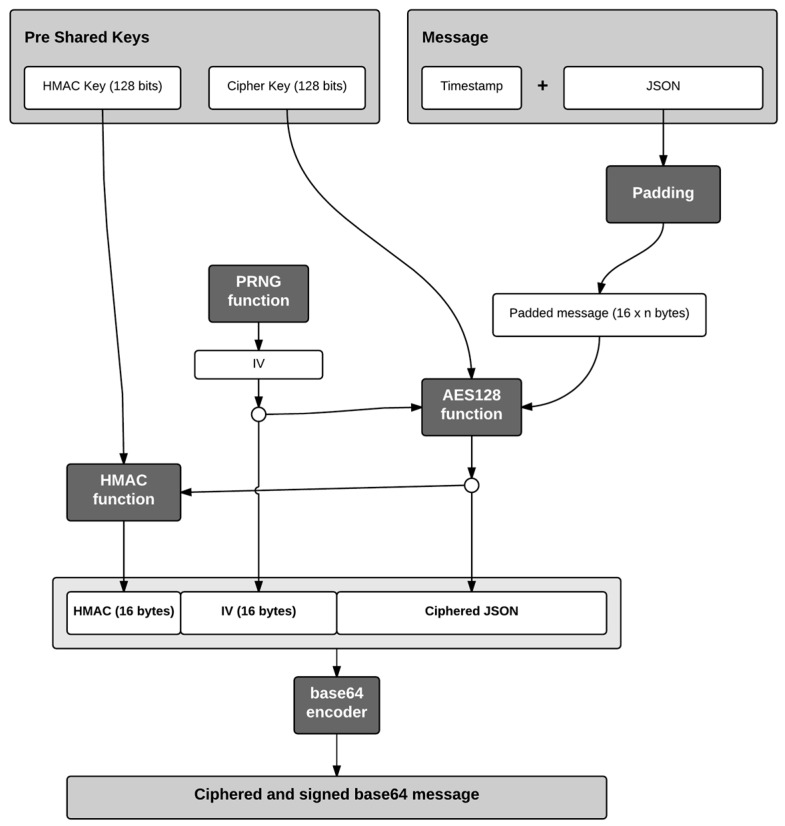
Generation of the encrypted message.

In our API RESTful design, sensitive information is only transmitted in the body of the HTTP requests so it has been decided to encrypt the JSON messages located on the body. After making these decisions, all the steps to encrypt the original message and validate it with the HMAC function are showed in the Figure 7. In Figure 7 the following protocol is established to protect communications between Arduino and the SLIM server:

1. SLIM Arduino server shares two private keys: one for the HMAC function and one for the encryption algorithm. Our IoT devices are resource-constrained. It has been discarded to implement a key exchange protocol session. Therefore these keys are pre-set in advance, both in the SLIM Proxy and in the IoT device.
2. Any message sent must attach a timestamp in the message body JSON. This decision is made for two reasons: first, it allows our IoT device to be synchronized with an external time reference despite it not always having an internal clock, and second and more importantly, it avoids man in the middle (MiM) attacks via a message replacement. To explain this type of attack let’s suppose, for example, an http request to transfer money. The body of this call (if we do not add a timestamp) will have a JSON message, such as: {“credit”: 2000}, which indicates 20 Euros. By encrypting this body, since the key is always the same, the encrypted message will be always the same. This will invite to a hypothetical hacker to perform the following operation:
  - Make a monetary transaction for a very high amount and intercepts the encrypted message (eavesdropping).
  - Back and perform an operation for a very low amount, then intercept the message and replace it by the one intercepted previously with a higher amount.
  - The SLIM server will receive the second message with the body of the first, and once correctly decrypted, the monetary transaction will be executed with the high amount.
  As it can be seen for this type of attack, it is not necessary that the attacker be able to decrypt the message, it is enough to replace it. By adding a timestamp, we pseudo-randomize the encrypted message and once decrypted we know at what time the message was sent, so in the above example, the SLIM server will discard the second message for being too old.
3. Because messages can be of variable length, a fill (padding) to reach a length which is a multiple of the encryption algorithm block size has to be applied; in this case, a multiple of 16 bytes. There are different standards that determine how to apply padding to a plain text:
  - Space padding, which consist to fill with spaces.
  - ISO/IEC 7816 which determines that the first byte of the fill should be 0x80 (128 in decimal value) and the remaining bytes are filled with the value 0x00.
  - Public Key Cryptography Standards (PKCS7), which states that each padding bytes should have, as value, the number of bytes to be filled. For example if five bytes are needed, those 5 bytes will have the value 0x05.
  - ANSI X.923, indicating that all the padding bytes must have the value 0x00, except the last, whose value is the number of bytes to be filled.
  - ISO/IEC 9797 is not set as a standard for encryption algorithms but used for hash and MAC functions. In this case, all the padding bytes have the value 0x00.
4. The use of either mechanism can influence depending on the original text. It is important to avoid ambiguity decrypting the message because there may be problems in distinguishing padding from message bytes. This is especially important when the original message is in binary as any value is possible in a byte. In our particular case the messages to encrypt are in plain text, and they have a clear defined structure: a JSON string begins with left brace and ends with right brace, so the choice of any standard is not particularly relevant. Finally, although in the SLIM server all available methods are deployed, the ISO/IEC 9797 has been chosen because is the easiest to implement on the IoT device.
5. Once the message has filled, the encryption algorithm is applied to the message using the key shared between sender and receiver. The encryption mode (Cipher Block Chaining or CBC) requires an initialization vector (IV), which is generated randomly.
6. The resulting encrypted text message is sent to the HMAC (hash message authentication code) function together with the key shared between the IoT device and the SLIM server.
7. To the output of the HMAC function the initialization vector is added, which is needed to decrypt the message at destiny and also the encrypted message.
8. Finally, since the generated message is in binary format, a Base64 encoding is applied to represent it by ASCII characters. This process consists of dividing the binary message in blocks of 6 bits and assigning to each of these blocks (which may have 64 different values) a character. The characters used are uppercase letters, lowercase letters, numeric digits and the symbols (+) and slash (/). On the receiver side it is necessary to perform the same steps in reverse order to obtain the original message.

## 7. Call Flow Scenario

**Figure 8:**
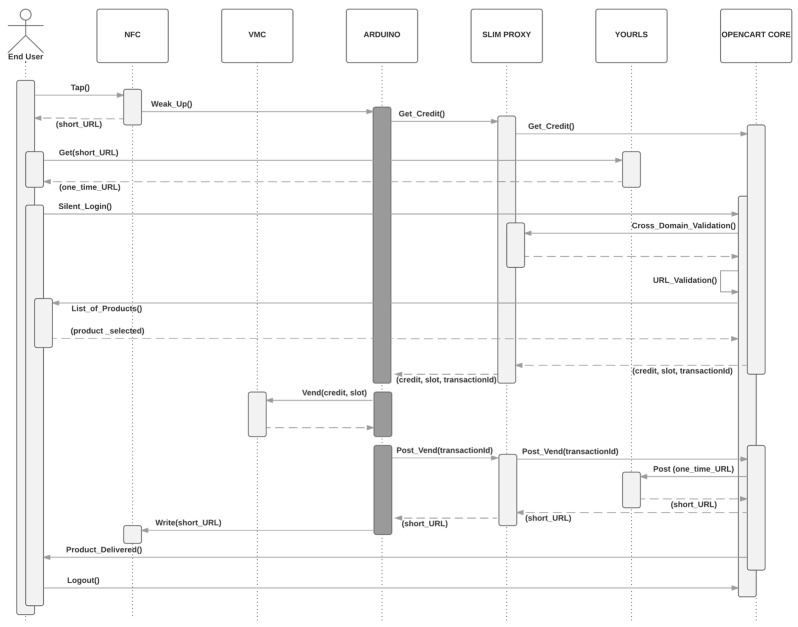
Call flow example of usage of one-time URL in a vending machine.

In the Section 5, the underlying cloud architecture is presented with the aim to describe the main building blocks involved in the tokenization of URLs. Besides, in the Section 6, a silent multi-domain logging mechanism was introduced to secure the access to the generated one-time URLs. Figure 8 shows a call flow diagram of the process of ordering a product in our retrofitted vending machine (see Figure 1). This diagram combines the different mechanisms introduced in this paper.

The scenario starts when the end user provided with a NFC-enabled smartphone taps the NFC module (see Figure 1). The NFC module will launch an interruption to Arduino (see Figure 1) to let him know that someone is reading the short URL. Then, Arduino will query the shopping cart of Opencart (see Figure 4) and wait for the details of the ordered product. Section 5.1.2 presents all the communications between Arduino and Opencart. These communications are routed through the SLIM Proxy (see Figure 4).

Coming back to the first step, when the end user taps the NFC module, the browser of the smartphone will retrieve the one-time URL from YOURLS, the URL shortener (see Figure 4) and automatically, logging into the vending machine webapp as it is described in the Figure 7. The access to the webapp will be granted with the cross-domain mechanism described in the Section 6.2.

In the second step, the end user will buy online the desired product selecting it on the touch screen of his smartphone (see demo [14]) and OpenCart will reply to Arduino, which was waiting for the ordered product details. Arduino will starts a dialogue with the vending machine controller (VMC) (see Figure 1.) and dispense the ordered product. Arduino will confirm to OpenCart that the product has been delivered and will retrieve a new one-time URL from the server which will be finally announced through the NFC module. As the final step, the end user will be notified on his smartphone that the product has been delivered and the web session will be closed.

## 8. Open Hardware Prototype and Preliminary Benchmarks

**Figure 9:**
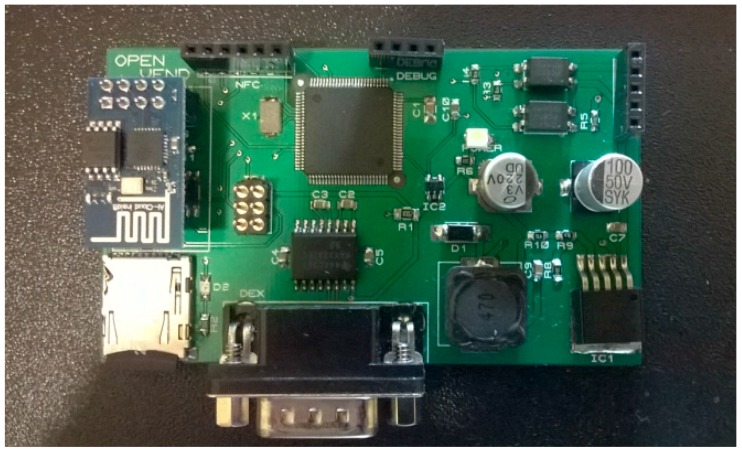
Arduino Mega compatible prototype for vending machines.

Arduino compatible electronic components introduced in Section 3 have been selected to build our open hardware prototype. As reference, Figure 9 shows one of our bespoke Arduino Mega open hardware design. This board is powered by the Multidrop Bus Standard (MDB) interface of the vending machine and it is able to communicate with our cloud solution via WiFi, and to retrieve the one-time URL which is announced via NFC.

To retrofit a vending machine we need a plug-and-play device that can be turned ON and OFF at any time. Arduino is a simple microcontroller which does not have to be properly shut down before turning OFF the power. Arduino is also well suited for repeated type of works like keep alive the slave-master synchronization mechanism, which requires sending every 200 ms acknowledge messages to the vending machine controller. As disadvantage, Arduino is not capable of doing multiple tasks at a time, like a computer as Raspberry Pi does. To overcome such limitations we programed a pseudo multitask mechanisms consisting of:

(a) Interrupts every 200 ms, to keep alive the master-slave synchronization with the VMC.
(b) Finite-State Machine (FSM) computation models, to handle the call flows with the back-end servers and to interact with other peripheral devices such as NFC modules, hacked vending machine keypad, etc.

To improve the response time using FSM handlers, it is important not to lose cycles of microcontroller, therefore we avoided the usage of the directive delay(). Unfortunately this is a directive commonly used in Arduino libraries, forcing us to discard them and code at a very low level. These obstacles become benefits thanks to Arduino community best practices. We learned how to optimize and control the memory of our code, resulting in a firmware below the 60 kilobytes, and a stable dynamic memory allocation of 6331 bytes. These facts are observed after running a stress test of three hundred consecutive orders, as shown the Table 2. These achievements ensured the robustness of our open hardware design.

The GET, VEND and POST rows of Table 2 refer respectively to Get_Credit(), Vend(credit,slot) and Post_Vend(transactionId) calls initiated by Arduino as Figure 8 shows. Two main calls are required to complete an order. First, Arduino performs a GET RESTful call and once the product has been selected on the smartphone, Arduino gets the ordered product details, including the price and the slot on which the product is located in the vending machine. Gathering such info takes 2972 ms. At this moment the vending machine performs the VEND in 1499 ms and the product is delivered. In other words, the product is delivered in 4471 ms providing a good purchasing experience. To complete the ordering cycle, a second POST RESTful call is performed to update the system with the renewed URL and to inform the user that the order has been successfully been delivered. The POST query requires an additional 4565 ms, which overlaps with the time used by the consumer to collect the product from the vending machine. As outcome, the overall cycle is completed in 9049 ms and the system is ready to serve new orders with the renewed one-time URL.

## 9. Conclusions

The great potential of the Internet of Things (IoT) is widely known [15]. To unlock its full potential in order to develop IoT solutions it is necessary to bring together connected devices and web standards, conforming the new paradigm of the Web of Things (WoT).

The initial motivation of this research was to provide an alternative mobile payment for unattended point of sales just using web standards technologies, without the need to install any native application or mobile wallet. As payment systems deal with sensitive and private data, it makes security to be an integral part of presented work. This paper presents a proximity application layer security mechanism for the WoT applied to unattended point of sales. It is shown the complete design of the secured proximity layer system to interact with connected things from mobile devices. This approach includes cryptography and tokenization for low-resource devices to ensure its security. Specifically the mechanism to tokenize the URLs, which uniquely identify the thing with its digital avatar. These URLs are broadcasted through pervasive short range radio-frequency technologies available on smartphones.

It has also been detailed how the system developed has been integrated as a part of an end-to-end cloud architecture for unattended point of sales to enable proximity mobile payments. At this point, it is worth it to mention a successful payment mechanism based on tokenized QRs that WeChat [16] has introduced recently in China. Conceptually the presented one-time URLs can be converted to tokenized QRs and be easily displayed in any machine with a screen. Reading these QRs will point consumers to our one-time URLs, so possibilities are endless for disrupting the mobile payment ecosystem of unattended point of sales and by extension to any point of sales.

Moreover, other aspects of the security system developed have been analyzed. These aspects include encryption of the user credentials on mobiles, the verification of multiple origins and a two factor authentication mechanism among others. Standardized secured mechanisms of communication have been used always looking for availability of open source libraries. 

Finally, some performance benchmarks have been presented to validate the robustness of our open hardware designs and the overall purchasing experience. The next step in this project would be to demonstrate the benefits of our approach in a real case scenario.

## Figures and Tables

**Table 1:** AES Processing time with Arduino Mega.

Text to Encript (bytes)	Key Length	Operation	Processing Time (s)
16	128	Encryption	0.51
16	128	Decryption	0.59
16	192	Encryption	0.65
16	192	Decryption	0.69
16	256	Encryption	0.72
16	256	Decryption	0.78

**Table 2:** Performance test result.

Test	Count	Minimum (ms)	Maximum (ms)	Average (ms)
Cycles	300	7918	13,457	9049
GET	295 *	2235	5189	2972
VEND	295 *	1499	1501	1499
POST	295 *	4140	6994	4565
FREE Memory Allocation	300	6331	6331	6331

* Difference due to 5 timeouts from server.

## Appendix

Below is the source code for the AES performance test showed in Table 1 on Arduino Mega. The library used can be downloaded from github [17].

**Algorithm 1: Source code for the AES algorithm on Arduino Mega**
#include <AESLib.h> 1: void setup(){
2: Serial.begin(57600);
3: uint8_t key[] = {0,1,2,3,4,5,6,7,8,9,10,11,12,13,14,15};
4: char data[] = "0123456789012345"; //16 chars == 16 bytes
5: aes128_enc_single(key, data);
6: Serial.print("encrypted:");
7: Serial.println(data);
8: aes128_dec_single(key, data);
9: Serial.print("decrypted:");
10: Serial.println(data);
11: }
12: void loop() {}
